# Z-Scheme ZIF-8/Ag_3_PO_4_ Heterojunction Photocatalyst for High-Performance Antibacterial Food Packaging Films

**DOI:** 10.3390/ma18112544

**Published:** 2025-05-28

**Authors:** Qingyang Zhou, Zhuluni Fang, Junyi Wang, Wenbo Zhang, Yihan Liu, Miao Yu, Zhuo Ma, Yunfeng Qiu, Shaoqin Liu

**Affiliations:** 1 Faculty of Life Science and Medicine, School of Medicine and Health, Harbin Institute of Technology, Harbin 150080, China; 2022112312@stu.hit.edu.cn (Q.Z.); 2022112282@stu.hit.edu.cn (J.W.); 2022112300@stu.hit.edu.cn (W.Z.); 2022112311@stu.hit.edu.cn (Y.L.); shaoqinliu@hit.edu.cn (S.L.); 2 Faculty of Life Science and Medicine, School of Life Science and Technology, Harbin Institute of Technology, Harbin 150001, China; zfang034@connect.hkust-gz.edu.cn (Z.F.); zhuoma@hit.edu.cn (Z.M.)

**Keywords:** ZIF-8, Ag_3_PO_4_, Z-scheme photocatalysis, antibacterial film, food packaging

## Abstract

Food spoilage caused by microbial contamination remains a global challenge, driving demand for sustainable antibacterial packaging. Conventional photocatalytic materials suffer from limited spectral response, rapid charge recombination, and insufficient reactive oxygen species (ROS) generation under visible light. Here, a Z-scheme heterojunction was constructed by coupling zeolitic imidazolate framework-8 (ZIF-8) with Ag_3_PO_4_, achieving dual-spectral absorption and spatial charge separation. The directional electron transfer from Ag_3_PO_4_’s conduction band to ZIF-8 effectively suppresses electron-hole recombination, prolonging carrier lifetimes and amplifying ROS production (·O_2_^−^/·OH). Synergy with Ag^+^ release further enhances bactericidal efficacy. Incorporated into a cellulose acetate matrix (CAM), the ZIF-8/Ag_3_PO_4_/CAM film demonstrates 99.06% antibacterial efficiency against meat surface microbiota under simulated sunlight, alongside high transparency. This study proposes a Z-scheme heterojunction strategy to maximize ROS generation efficiency and demonstrates a scalable fabrication approach for active food packaging materials, effectively targeting microbial contamination control and shelf-life prolongation.

## 1. Introduction

Increasing consumer demand for premium food products has amplified requirements for advanced antibacterial technologies to address microbial contamination, a persistent global challenge compromising food safety and shelf-life extension [1]. Active packaging systems, particularly antibacterial films, have gained prominence through the incorporation of functional agents into polymeric matrices to inhibit microbial growth via controlled release mechanisms or surface-mediated interactions. Although conventional strategies employing quaternary ammonium compounds or natural extracts show moderate antibacterial performance, their drawbacks—including limited antimicrobial spectra, environmental persistence, and potential toxicity—highlight the necessity for sustainable alternatives. Crucially, excessive antibiotic usage in food packaging raises significant concerns about aquatic ecosystem contamination and antimicrobial resistance evolution, highlighting the imperative need for nanotechnology-enabled approaches that balance high efficacy with a reduced environmental footprint [2].

Recent advancements in nanomaterial engineering have unveiled metallic and photocatalytic systems as promising candidates for antibacterial applications [3]. Silver-based nanomaterials, leveraging their broad-spectrum biocidal activity via Ag^+^ ion release and oxidative stress induction, have dominated research efforts. For instance, studies have demonstrated silver nitrate nanocomposites with potent activity against *Staphylococcus aureus* (*S. aureus*) and *Escherichia coli* (*E. coli*), while others enhanced alginate films using silver nanoparticles (AgNPs) and plant-derived oils for multi-pathogen inhibition [4]. Despite these successes, standalone Ag nanomaterials face challenges such as aggregation-induced performance decay and insufficient reactive oxygen species (ROS) generation under visible light. Metal–organic frameworks (MOFs), exemplified by zeolitic imidazolate framework-8 (ZIF-8), offer a compelling alternative due to their tunable porosity, high surface area, and intrinsic antibacterial properties [5]. Under solar irradiation, ZIF-8 facilitates electron transfer to activate molecular oxygen, generating superoxide radicals (·O_2_^−^) and hydrogen peroxide (H_2_O_2_) for bacterial inactivation. However, its wide bandgap (~3.3 eV) restricts light absorption to UV regions, while rapid electron-hole recombination further curtails photocatalytic efficiency and ROS yield-critical bottlenecks, hindering practical implementation.

To address these limitations, Z-scheme heterojunction systems have gained attention for their ability to synergize complementary photocatalysts, thereby broadening the spectral response [6] and suppressing charge recombination [7]. Inspired by natural photosynthesis, such configurations enable spatial separation of redox centers, prolonging carrier lifetimes and amplifying ROS production [8]. This study innovatively couples ZIF-8 with Ag_3_PO_4_, a visible-light-responsive semiconductor (bandgap ~2.34 eV), to construct a dual-functional Z-scheme system [9,10]. The heterojunction not only extends light harvesting to UV-vis regions [11] but also facilitates directional electron transfer from Ag_3_PO_4_ to ZIF-8, effectively decoupling photogenerated charges while leveraging Ag^+^ release for synergistic bactericidal effects [12].

Despite advancements in photocatalytic antimicrobial materials, significant challenges remain in transitioning laboratory-scale innovations into food-grade packaging systems at scalable levels. There has been insufficient research focused on optimizing ROS production under real-world storage environments, which is a critical determinant of antibacterial effectiveness in perishable food matrices. Herein, we bridge these gaps by engineering a cellulose acetate matrix-embedded ZIF-8/Ag_3_PO_4_ composite film that displays high photocatalytic activity [11,13], thereby offering a scalable platform for active food packaging. Through systematic characterization and mechanistic studies, this work elucidates the interplay between heterojunction design, ROS dynamics [14], and antimicrobial performance, ultimately validating the composite’s potential to revolutionize food preservation technologies.

## 2. Materials and Methods

### 2.1. Materials

The following chemicals were purchased from Aladdin Biochemical Technology Co., Ltd. (Shanghai, China): zinc nitrate hexahydrate (Zn(NO_3_)_2_·6H_2_O), methanol (CH_3_OH), melamine (Melm), acetone (C_3_H_6_O), disodium hydrogen phosphate dihydrate (NaH_2_PO_4_·2H_2_O), silver nitrate (AgNO_3_), propidium iodide (PI), and syto9. Polyvinylpyrrolidone (PVP) and the polymeric compound (C_6_H_7_O_2_) (OOCCH_3_)_3n_ were obtained from Macklin Biochemical Co., Ltd. (Shanghai, China).

### 2.2. Characterization

The surface morphology and structure of ZIF-8 material were characterized by scanning electron microscopy (SEM, Quanta FEG, Thermo Fisher Scientific, Waltham, MA, USA) and X-ray diffraction (XRD, Rigaku D/Max 2500 PC, Rigaku Corporation, Tokyo, Japan). Additionally, the molecular structure was confirmed by Raman spectroscopy (Raman, inVia-Reflex, Renishaw plc, London, UK), demonstrating the successful synthesis of ZIF-8 material. Transmission electron microscopy (TEM, Talos F200C, Thermo Fisher Scientific, Waltham, MA, USA) was employed to observe the presence of black nanoparticles on the surface of ZIF-8 particles. High-resolution transmission electron microscopy (HRTEM, JEM-2100F, JEOL Ltd., Tokyo, Japan), selected area electron diffraction (SAED), and X-ray photoelectron spectroscopy (XPS, K-Alpha, Thermo Fisher Scientific, Waltham, MA, USA) further verified the successful composite of ZIF-8/Ag_3_PO_4_. Subsequently, the absorption characteristics of the material to different wavelengths of light were evaluated using a fluorescence spectrophotometer (SG-723, Shanghai INESA Analytical Instrument Co., Ltd., Shanghai, China). The types of free radicals were detected by electron paramagnetic resonance spectroscopy (EPR spectrometer, EPR-100, CIQTEK (Hefei) Technology Co., Ltd., Hefei, China). The permeability of bacterial cell membranes was tested using a microplate reader (Multiskan FC, Thermo Fisher Scientific, Waltham, MA, USA). Finally, live/dead bacterial staining was performed using an inverted fluorescence microscope (NIB950-FL, Ningbo Yongxin Optics Co., Ltd., Ningbo, Zhejiang, China) to verify the antibacterial properties of the material. The food safety of the composite film was confirmed by inductively coupled plasma optical emission spectrometry (ICP-OES, Avio 200, PerkinElmer Inc., Waltham, MA, USA).

### 2.3. Preparation of ZIF-8

Take 1.5 g of Zn(NO_3_)_2_·6H_2_O and dissolve it in 50 mL of methanol. Stir until completely dissolved. Then, take 3.3 g of 2-methylimidazole (Melm) and dissolve it in 50 mL of methanol, stirring until completely dissolved. Add the Melm solution to the Zn(NO_3_)_2_ solution and stir for 15 min at 1200 rpm. Allow the mixture to stand for 24 h to obtain the crystals. Finally, wash the precipitate with methanol and dry it at 60 °C.

### 2.4. Preparation of ZIF-8/Ag_3_PO_4_ Composite Material

Take 150 mg of ZIF-8 and 50 mg of PVP and add them to 50 mL of methanol. Sonicate for 10 min, then add 150 mg of silver nitrate to the mixture of ZIF-8 and PVP and stir for 20 min. Filter the mixture and redissolve the obtained samples in methanol. Take 150 mg of sodium dihydrogen phosphate and dissolve it in 40 mL of deionized water, then mix this solution with the previous one. Finally, filter again, wash with methanol, and dry at 60 °C to obtain ZIF-8/Ag_3_PO_4_.

### 2.5. Preparation of ZIF-8/Ag_3_PO_4_ Composite Film

Take 2 g of cellulose acetate and 10 mL of acetone in a centrifuge tube. Stir with a magnetic stirrer until completely dissolved. Pour the solution onto a glass petri dish, let it dry in a fume hood, then soak it in cold water to remove the dish and let it dry naturally or in an oven at a suitable temperature.

### 2.6. Antibacterial Experiments

The antibacterial performance was tested using the plate colony counting method. The antibacterial target was Gram-negative *E. coli*. First, use an inoculation loop to inoculate the preserved bacteria into an LB solid medium and incubate in a 37 °C incubator for 24 h. Take the grown colonies and inoculate them into LB liquid medium at a ratio of 1:15. Incubate in a constant temperature shaking incubator at 37 °C for 12 h, then dilute the bacterial solution with PBS to a concentration of 1 × 10^7^ CFU/mL. Mix 400 μL of the diluted bacterial solution with 20 μL of a 0.5 mg/mL aqueous solution of the powder sample. The weight concentration of ZIF-8/Ag_3_PO_4_ composite was 23.8 µg/mL. Incubate in a constant temperature shaking incubator at 37 °C for 3 h. One group of the incubated mixtures was exposed to simulated sunlight (using a xenon lamp) for 1 h, while the other group served as a control without exposure. Take 5 μL of the mixed solution, add it to 1 mL of PBS, and dilute it 500 times. Take 20 μL of the diluted solution and spread it on an agar plate, then incubate in a constant temperature incubator for 12 h. Count the number of colonies on the agar plates using a colony counter, and evaluate the antibacterial activity of the sample by calculating the antibacterial rate (%) as follows:

(1) $$\mathrm{Antibacterial}\;\mathrm{rate}=\frac{\mathrm{Number}\;\mathrm{of}\;\mathrm{colonies}\;\mathrm{in}\;\mathrm{the}\;\mathrm{control}\;-\;\mathrm{Number}\;\mathrm{of}\;\mathrm{colonies}\;\mathrm{in}\;\mathrm{the}\;\mathrm{sample}}{\mathrm{Number}\;\mathrm{of}\;\mathrm{colonies}\;\mathrm{in}\;\mathrm{the}\;\mathrm{control}}\times 100\%$$

The antibacterial performance was tested using the plate colony counting method, with Gram-negative *E. coli* as the antibacterial target. Inoculate the preserved bacterial strain into LB solid medium using an inoculation loop and cultivate in a 37 °C incubator for 24 h. Subsequently, inoculate the growing bacterial colonies into LB liquid medium at a ratio of 1:15 and cultivate in a constant temperature shaking incubator at 37 °C for 12 h. Dilute the bacterial suspension with PBS to a concentration of 1 × 10^7^ CFU/mL. Cut the ZIF-8/Ag_3_PO_4_ composite film into small pieces of 2 cm^2^, place into the diluted bacterial suspension, and incubate. Divide the incubated mixture into two groups: expose one group to light from a xenon lamp simulating AM1.5G sunlight for 1 h, while the other group serves as a control without any treatment. After removing the composite film, place it in 1 mL of PBS and further dilute 10^3^ times. Plate a 20 μL aliquot of the diluted suspension onto a culture dish, then incubate it upside down in a constant temperature incubator for 12 h. Finally, count the number of colonies on the culture dishes using a colony counter to evaluate the antibacterial rate using Formula (1).

### 2.7. Practical Application of Antimicrobial Food Packaging Film

Cut fresh pork tenderloin into uniform small pieces of 5 cm^2^. Wrap one group with ZIF-8/Ag_3_PO_4_ composite antimicrobial food packaging film (two pieces); leave the other group untreated (two pieces). Within each group, expose one piece to light from a xenon lamp simulating AM1.5G sunlight; leave the other piece not exposed to light. Place the samples in a simulated real food storage environment for 3 days. After this period, extract the surface microbiota and dilute it 10^3^ times. Plate a 20 μL aliquot of the diluted suspension onto a culture dish and incubate in a constant temperature incubator for 12 h. Finally, count the number of colonies on the culture dishes using a colony counter to evaluate the antibacterial activity of the sample through the antibacterial rate (%) using formula (1).

## 3. Results and Discussions

### 3.1. ZIF-8/Ag_3_PO_4_ Composite Preparation and Characterization

**Figure 1:**
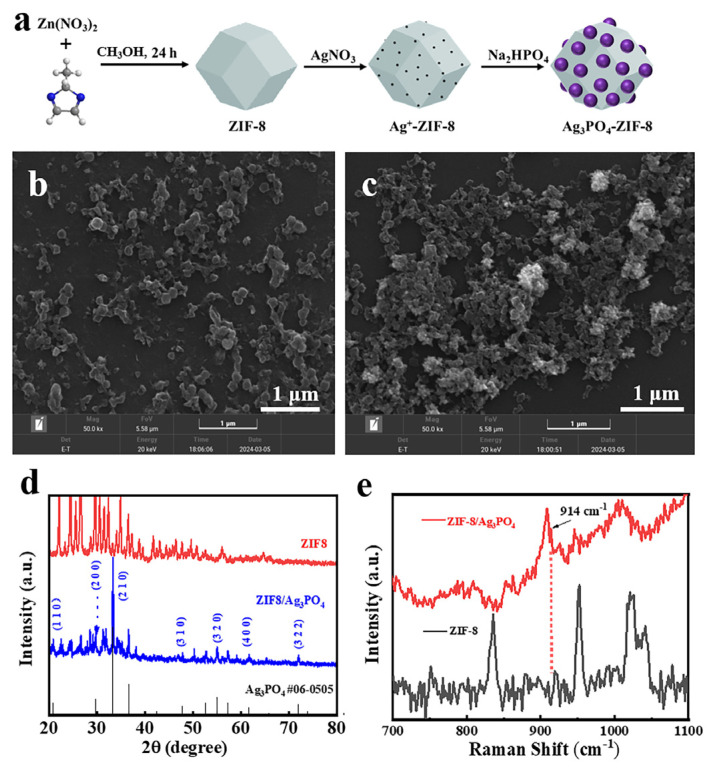
(**a**) Schematic illustration of the synthesis process; SEM images of (**b**) ZIF-8 and (**c**) ZIF-8/Ag_3_PO_4_, respectively; (**d**) XRD patterns of ZIF-8 and ZIF-8/Ag_3_PO_4_; (**e**) Raman spectra of ZIF-8 and ZIF-8/Ag_3_PO_4_.

Figure 1a depicts the synthesis process of the ZIF-8/Ag_3_PO_4_ composite material. SEM investigation (Figure 1b) indicated that the ZIF-8 particles possessed an average diameter of around 150 ± 18 nm. This result correlates with established ZIF-8 size distributions, confirming the synthetic reproducibility in particle dimensions and structural characteristics. Furthermore, SEM characterization of the ZIF-8/Ag_3_PO_4_ composite demonstrated increased average particle sizes (185 ± 25 nm), as seen in Figure 1c, resulting from Ag_3_PO_4_ nanoparticle deposition on ZIF-8 surfaces.

The XRD pattern of the ZIF-8 material (Figure 1c) displays distinct diffraction peaks that align closely with standard reference data (CCDC No. 734623) [15,16], hence validating the effective synthesis of ZIF-8 with a methanol-based solvent solution. The XRD pattern of the ZIF-8/Ag_3_PO_4_ composite material exhibits the characteristic peaks of Ag_3_PO_4_ at 20.88°, 33.56°, 36.36°, 47.79°, and 55.02°, which correspond to the (110), (200), (210), (310), and (320) crystal planes, respectively [17,18,19]. The lack of impurity peaks in the XRD pattern confirms the successful synthesis of a high-purity composite material, with Ag_3_PO_4_ effectively integrated into the ZIF-8 crystalline framework to create a heterostructured composite.

Raman spectra were obtained with a laser excitation wavelength of 785 nm [20]. Figure 1d illustrates that the Raman spectra of the ZIF-8/Ag_3_PO_4_ composite have a novel peak at 914 cm^−1^ compared with ZIF-8, indicative of the symmetrical stretching vibration of the [PO_4_] cluster in Ag_3_PO_4_ [21]. This discovery demonstrates that the vibrational modes of Ag_3_PO_4_ were successfully preserved and are distinctly seen in the composite material.

The integrated results from the SEM, XRD, and Raman spectroscopy investigations confirm the effective synthesis of the ZIF-8/Ag_3_PO_4_ composite material. The synthesized composite has high purity, accurate structural attributes, and superior crystallinity, establishing a solid basis for its prospective applications in advanced material systems.

**Figure 2:**
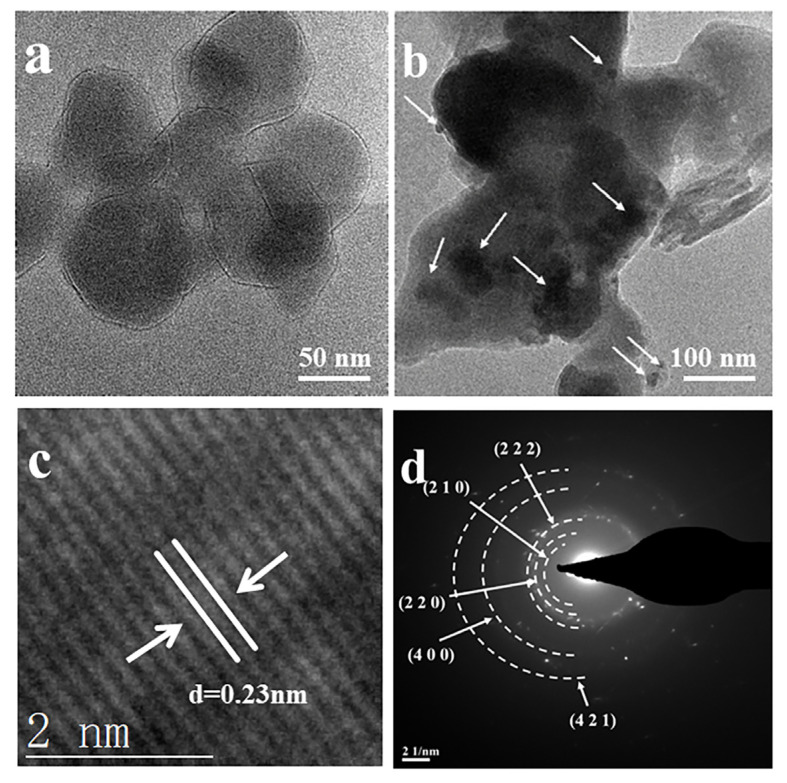
TEM images of (**a**) ZIF-8 and (**b**) ZIF-8/Ag_3_PO_4_, (**c**) HRTEM image of ZIF-8/Ag_3_PO_4_, and (**d**) SAED pattern of ZIF-8/Ag_3_PO_4_. Arrows in (**b**) indicated the presence of Ag_3_PO_4_ nanoparticles.

ZIF-8 particles exhibit smooth morphologies, as seen in Figure 2a. Conversely, the TEM analysis revealed surface-deposited, black-spotted nanoparticles (white arrows, Figure 2b) on ZIF-8 substrates, assigned to Ag_3_PO_4_ nanocrystals. High-resolution transmission electron microscopy (HRTEM) and selected area electron diffraction (SAED) were performed to confirm this. HRTEM revealed a lattice spacing of 0.23 nm (Figure 2c), which corresponds to the distinctive spacing of Ag_3_PO_4_. The SAED pattern displays clear polycrystalline diffraction rings (Figure 2d), indexed to the (210), (220), and (222) planes of Ag_3_PO_4_ [22]. These findings align well with the XRD data for Ag_3_PO_4_. The HRTEM and SAED investigations collectively validate the effective synthesis of Ag_3_PO_4_ nanoparticles on the ZIF-8 surface, offering structural insights into the composite material.

**Figure 3:**
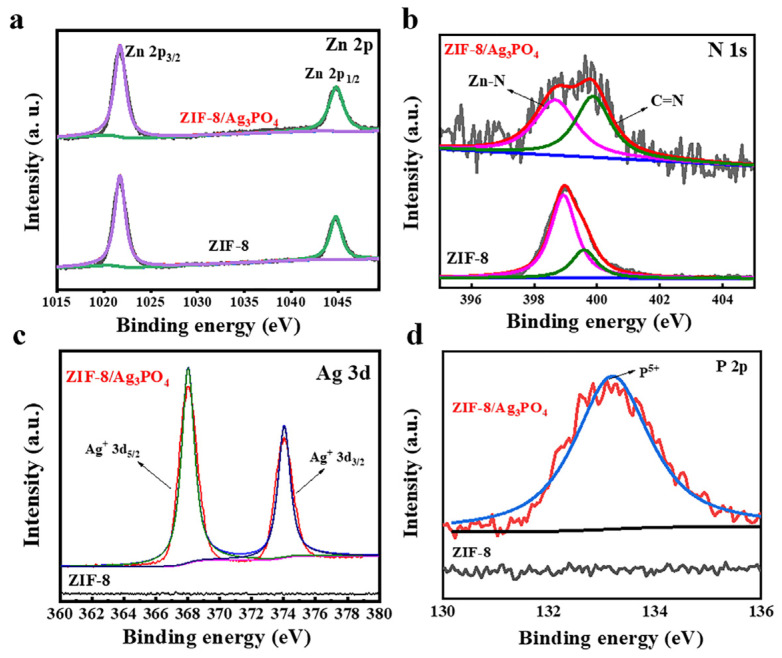
High-resolution XPS spectra of (**a**) Zn 2p, (**b**) N 1s, (**c**) Ag 3d, and (**d**) P 2p.

XPS analysis confirmed the presence of Zn, O, C, N, Ag, and P, consistent with the expected composition of the composite material (Figure S1). The high-resolution Zn 2p spectra (Figure 3a) display peaks at 1044.95 eV (Zn 2p_1/2_) and 1021.87 eV (Zn 2p_3/2_), characteristic of Zn in the ZIF-8 framework. The N 1s spectra (Figure 3b) exhibit peaks at 398.8 eV and 399.9 eV, corresponding to Zn-N and C=N bonds, respectively. The Zn-N bond arises from the coordination between Zn and N in the imidazole ligand. In contrast, the C=N bond is attributed to the organic backbone of the framework, confirming the structural integrity of ZIF-8.

The Ag 3d XPS spectra (Figure 3c) identify peaks at 373.93 eV and 367.98 eV, assigned to the spin-orbit coupling peaks of Ag^+^, confirming the oxidation state of Ag. The peak at 134.6 eV (Figure 3d), attributed to P^5+^ in Ag_3_PO_4_, provides strong evidence supporting the formation of Ag_3_PO_4_. Collectively, these findings validate the successful incorporation of Ag_3_PO_4_ into the ZIF-8 matrix, offering insights into the composite’s chemical composition and bonding properties.

**Figure 4:**
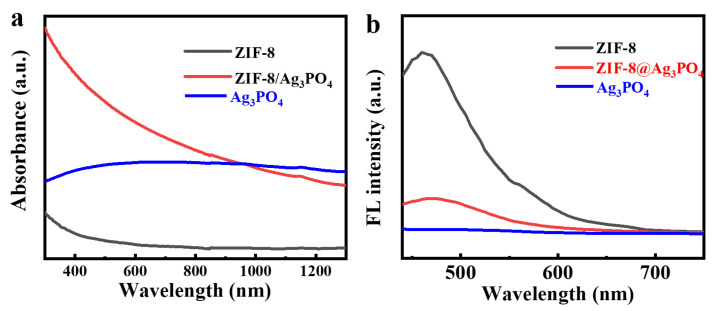
(**a**) UV-Vis-NIR diffuse reflectance spectra of ZIF-8, Ag_3_PO_4_, and ZIF-8/Ag_3_PO_4_, respectively. (**b**) Fluorescence emission spectra of ZIF-8, Ag_3_PO_4_, and ZIF-8/Ag_3_PO_4_, respectively.

In photocatalytic applications, a material’s capacity to absorb light directly influences its catalytic efficacy and antibacterial properties. The UV-Vis-NIR diffuse reflectance spectrum of the ZIF-8/Ag_3_PO_4_ composite (Figure 4a) demonstrates markedly improved visible light absorption over ZIF-8. This absorption capacity emphasizes the composite’s ability to effectively capture solar energy over a broad spectra range, while also showcasing its optimized electronic structure and enhanced optoelectronic characteristics. The improved light absorption is anticipated to significantly boost the composite’s photocatalytic and antibacterial efficacy.

Utilizing an excitation wavelength of 400 nm, the fluorescence emission spectrum (Figure 4b) demonstrates a significant decrease in the fluorescence intensity of the ZIF-8/Ag_3_PO_4_ composite relative to that of ZIF-8 alone. This decrease signifies that the composite efficiently inhibits electron-hole recombination. In photocatalytic processes, the recombination of photogenerated charge carriers diminishes overall photocatalytic efficiency by depleting absorbed light energy and reducing the availability of charge carriers for redox reactions. These results demonstrate that the formation of a heterojunction significantly suppresses electron-hole recombination, hence prolonging the lifespan of photogenerated charge carriers. This extension enables an increased migration of charge carriers to the material’s surface, thus promoting redox reactions with adsorbed molecules. As a result, an increased production of ^1^O_2_ or ·OH occurs, improving the photocatalytic effectiveness of the material.

### 3.2. Detection of Reactive Oxygen Species

**Figure 5:**
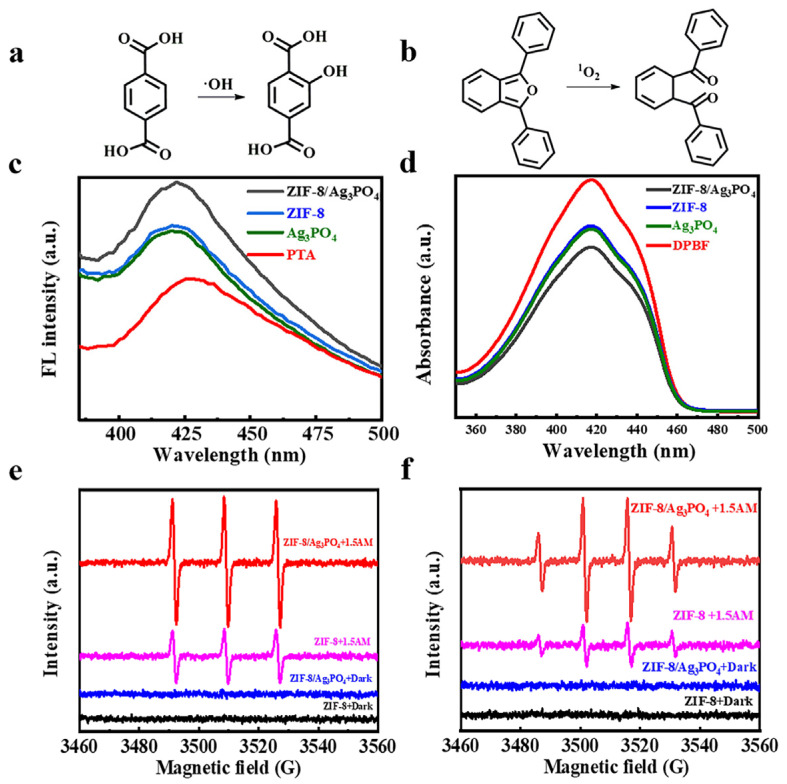
(**a**) PTA for the detection of ·OH, (**b**) DPBF for the detection of ^1^O_2_, (**c**) fluorescence spectrum of PTA, (**d**) UV-Vis spectrum of DPBF, and EPR analysis for (**e**) ·OH and (**f**) ^1^O_2_.

Terephthalic acid (PTA) was employed as a molecular probe for hydroxyl radical (·OH) detection (Figure 5a,c). Upon reaction with ·OH, PTA is selectively converted to 2-hydroxyterephthalic acid, a fluorescent derivative exhibiting characteristic emission. ·OH concentrations were quantified through spectrofluorometric intensity measurements. Upon light irradiation, the ZIF-8/Ag_3_PO_4_ nanocomposite exhibited a markedly enhanced fluorescence intensity at 425 nm relative to ZIF-8 and Ag_3_PO_4_ individually, signifying an increased generation of ·OH. The increased production of ·OH underscores the composite’s ability to boost photothermal antibacterial efficacy.

1,3-Diphenylisobenzofuran (DPBF) was utilized as a selective probe for the detection of singlet oxygen (^1^O_2_) (Figure 5b,d). DPBF selectively interacts with ^1^O_2_ to generate an endoperoxide, which then decomposes into 1,2-dibenzoylbenzene. The characteristic renders DPBF a proficient indication for identifying ROS [23]. Upon interaction with ^1^O_2_, DPBF experiences irreversible oxidation, resulting in a fast reduction of its UV-Vis absorption intensity at 410 nm. Experimental results indicate that, under the same light irradiation conditions, the ZIF-8/Ag_3_PO_4_ composite produces a significantly higher decrease in the 410 nm absorption intensity than ZIF-8 and Ag_3_PO_4_. ZIF-8 and Ag_3_PO_4_ exhibit a reduction in absorption at 410 nm compared to the control; however, the decline is much less significant than that of the composite. This demonstrates that the ZIF-8/Ag_3_PO_4_ combination may produce elevated concentrations of ^1^O_2_ during the same illumination duration.

EPR spectroscopy is a powerful method for the direct identification of radicals with unpaired electrons, providing essential information regarding their kinds, concentrations, and structural properties. This work utilized 5,5-dimethyl-1-pyrroline-N-oxide (DMPO) as a spin-trapping agent to verify the existence of ·OH (Figure 5e). The interaction between DMPO and ·OH yields an adduct with a characteristic 1:2:2:1 quartet pattern in the EPR spectrum, offering compelling evidence for the significant generation of ·OH under the designated photocatalytic circumstances. Furthermore, 2,2,6,6-tetramethyl-4-piperidone hydrochloride (TEMP) serves as a probe for the detection of ^1^O_2_, producing a distinctive 1:1:1 triplet signal in the EPR spectrum (Figure 5f), thus corroborating the substantial production of ^1^O_2_. The thorough EPR investigation definitively confirmed the capacity of the ZIF-8/Ag_3_PO_4_ composite to generate ROS, hence creating a robust basis for its improved photocatalytic antibacterial efficacy under specified conditions.

### 3.3. Assessment of Membrane Permeability

The permeability of bacterial inner membranes was assessed utilizing o-nitrophenyl-β-D-galactopyranoside (ONPG) as a molecular probe. Following bacterial membrane disruption, ONPG may easily infiltrate the cell membrane and access the intracellular milieu, where it is degraded by β-D-galactosidase to yield o-nitrophenol (ONP), a compound that displays a distinctive yellow hue [24]. ONP exhibits considerable absorbance in the 410–420 nm wavelength region under alkaline circumstances, with the absorbance intensity at 420 nm being directly proportional to the extent of membrane permeability. This offers a dependable quantitative assessment of bacterial membrane integrity and permeability.

**Figure 6:**
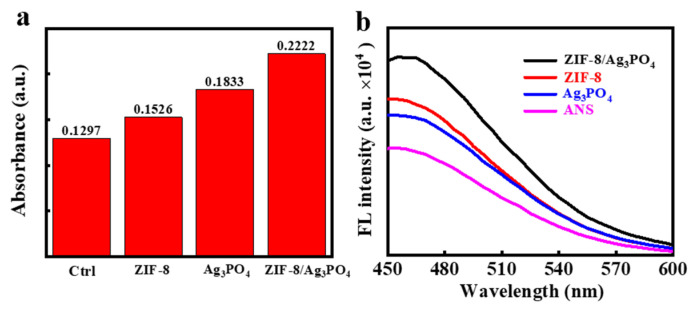
(**a**) Inner membrane and (**b**) outer membrane permeability of the control group (without the addition of any antibacterial materials), ZIF-8, Ag_3_PO_4_, and ZIF-8/Ag_3_PO_4_; of ZIF-8, Ag_3_PO_4_, and ZIF-8/Ag_3_PO_4_, respectively.

Figure 6a illustrates that the absorbance intensities at 420 nm for ZIF-8, Ag_3_PO_4_, and ZIF-8/Ag_3_PO_4_ nanocomposites are 0.1526, 0.1833, and 0.2222 (arbitrary units), respectively. The ZIF-8/Ag_3_PO_4_ nanocomposite demonstrates the greatest absorbance at 420 nm, signifying its enhanced ability to compromise the bacterial inner membrane. This improved permeability allows for better ONPG infiltration and subsequent hydrolysis, leading to increased ONP generation. These findings highlight the capacity of ZIF-8/Ag_3_PO_4_ to disrupt bacterial inner membrane integrity.

The permeability of the outer membrane of bacterial cells was evaluated utilizing 1-anilino-8-naphthalenesulfonic acid (ANS) as a fluorescent probe. ANS demonstrates diminished fluorescence intensity in aqueous solutions; nevertheless, when the bacterial membrane’s permeability increases, ANS infiltrates hydrophobic intracellular domains, resulting in a significant enhancement of fluorescence intensity. Figure 6b demonstrates that the fluorescence intensity of ZIF-8/Ag_3_PO_4_ under light irradiation is markedly greater than that of ZIF-8 or Ag_3_PO_4_ individually. This demonstrates that ZIF-8/Ag_3_PO_4_ significantly influences bacterial outer membrane permeability, facilitating more access for ANS molecules to the cell’s hydrophobic regions. The significant enhancement in fluorescence intensity indicates that ZIF-8/Ag_3_PO_4_ induces a stronger rupture of the outer membrane in *E. coli* than its constituents. The increased permeability of the outer membrane aligns with its overall higher antibacterial efficacy.

### 3.4. Microbial ATP Assay

**Figure 7:**
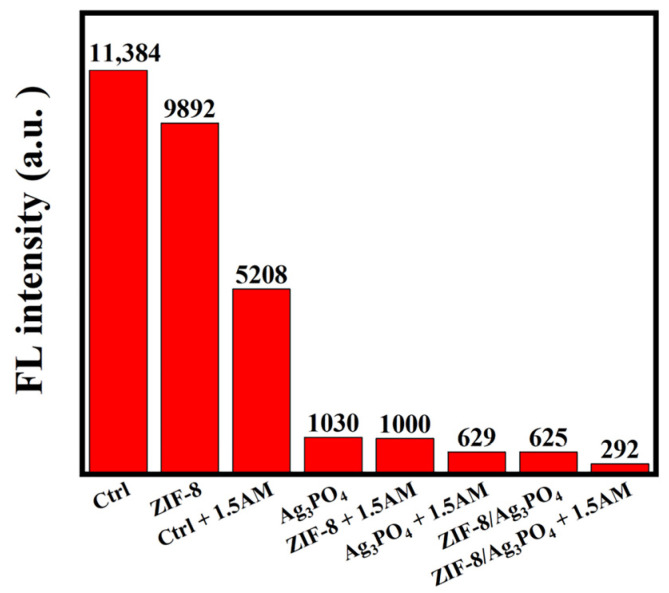
ATP content of control group without the addition any antibacterial materials (Ctrl), ZIF-8, Ctrl + 1.5AM, Ag_3_PO_4_, ZIF-8 + 1.5AM, Ag_3_PO_4_ + 1.5AM, ZIF-8/Ag_3_PO_4_, and ZIF-8/Ag_3_PO_4_ + 1.5AM.

Adenosine triphosphate (ATP) functions as a universal viability biomarker, with fluorescence intensity stoichiometrically correlating to ATP concentration for quantifying cellular metabolic activity and viability through fluorometric analysis [25]. This study utilized a fluorescence detector to measure emitted luminescence, offering information on the activity and prevalence of bacteria in the samples. The data analysis indicated that the ZIF-8/Ag_3_PO_4_ nanocomposite displays the lowest luminescent signal intensity (Figure 7), signifying the least ATP content among all evaluated samples. The findings indicate that the ZIF-8/Ag_3_PO_4_ composite significantly decreases the viable bacterial population. The results highlight the remarkable antibacterial efficacy of the ZIF-8/Ag_3_PO_4_ nanocomposite in inhibiting microbial proliferation.

### 3.5. Staining of Live/Dead Bacteria

**Figure 8:**
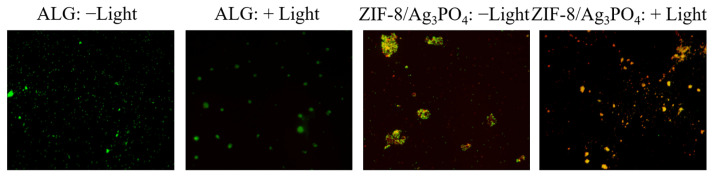
Live/dead *E. coli* bacterial staining images of the control group of ALG and ZIF-8/Ag_3_PO_4_.

The mechanism of live/dead bacterial labeling relies on the differential absorption of dyes by bacterial cells. Red dyes, including propidium iodide (PI), can infiltrate compromised cell membranes of deceased bacteria, but green dyes, such as syto9, can alone permeate the intact membranes of living bacteria. This facilitates a clear differentiation between viable and non-viable bacteria [26]. The photos indicate that cells in both the control group and the ZIF-8 group mostly display green fluorescence under light and dark circumstances, with negligible red fluorescence detected (Figure S3). In contrast, cells treated with Ag_3_PO_4_ exhibit yellow-green fluorescence under both conditions, whereas the ZIF-8/Ag_3_PO_4_ group displays significantly enhanced orange-red fluorescence (Figure 8), indicating more extensive bacterial membrane disruption and loss of viability. The results demonstrate that the ZIF-8/Ag_3_PO_4_ composite has enhanced antibacterial efficacy relative to ZIF-8 and Ag_3_PO_4_ individually. This observation aligns with the live/dead staining principle, where increased PI (orange-red) signal intensity correlates with compromised membrane integrity and non-viable bacterial populations.

### 3.6. Antibacterial Performance

**Figure 9:**
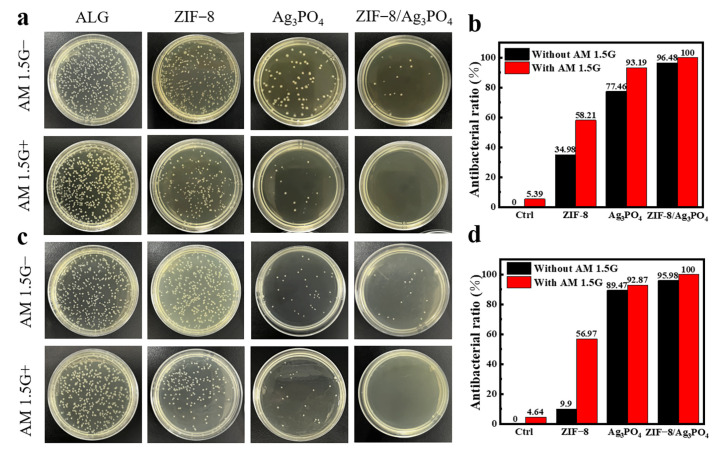
(**a**) *E. coli* colonies of powder materials under reaction conditions and simulated sunlight, (**b**) graphical representation of antibacterial efficiency of powder materials, (**c**) *E. coli* colonies of composite membrane materials under reaction conditions and simulated sunlight, and (**d**) graphical representation of antibacterial efficiency of composite membrane materials.

The samples to be tested were serially diluted in a gradient manner. A specific volume of the diluted sample solution was spread evenly onto petri dishes. The inoculated plates were then incubated under appropriate conditions for a specific period. After incubation, the number of colonies formed on the plates was counted. As shown in Figure 9a, both the powder materials and the CAM composite membrane materials demonstrate stronger antibacterial performance under simulated sunlight conditions compared with those in the dark reactions. Specifically, the antibacterial rate of Ag_3_PO_4_ reaches 93.19% in Figure 9b, while that of ZIF-8/Ag_3_PO_4_ reaches 100%. In previous studies, the PC/NMPs film made from natural melanin nanoparticles extracted from squid ink and natural pectin achieved a bactericidal rate of over 90% within 5 min [27]; polylactic acid bioplastics with added polyhexamethylene guanidine hydrochloride exhibited an antibacterial efficiency of 99.9% against *E. coli* in antibacterial packaging [28]. The chitosan-zinc oxide nanoparticle composite film demonstrated an antibacterial rate of approximately 95% against *E. coli* [29], while chitosan films embedded with silver nanoparticles showed an antibacterial rate of 90% to 99% against *E. coli* [30]. These findings suggest that the composite film in this study possesses favorable antibacterial properties.

The concentration-dependent antibacterial efficacy at 19.05 µg/mL was observed (Figure S4). The ZIF-8/Ag_3_PO_4_ composite achieves a 90.96% inhibition rate of *E. coli*, whereas 23.8 µg/mL (tested under identical conditions) induces complete eradication (100% inhibition of *E. coli*, Figure 9). This sharp transition suggests that the minimum bactericidal concentration lies between these concentrations, with 23.8 µg/mL serving as a provisional threshold for full bactericidal activity under the tested parameters.

Cellulose acetate, noted for its exceptional film-forming properties, biocompatibility, and biodegradability, is considered an optimal material for membrane production [31]. The combination of these properties and the extensive applicability in fields like biomedical devices and food packaging render cellulose acetate an exceptional prospect for composite membrane fabrication. Composite membranes of ZIF-8/Ag_3_PO_4_/cellulose acetate were fabricated with varying cellulose acetate concentrations (Figures S5 and S6). Membranes containing 20% cellulose acetate exhibit favorable properties among the evaluated formulations. This composition facilitates easy demolding and results in membranes with high optical transparency, indicating potential practical applicability. The ZIF-8/Ag_3_PO_4_ composite membrane exhibits superior antibacterial activity, achieving 100% inhibition under light irradiation and 95.98% inhibition in dark conditions in Figure 9c,d. Control groups reveal significantly reduced performance: the Ag_3_PO_4_ composite membrane demonstrates lower efficacy, with 92.87% inhibition under light and 89.47% in darkness. The ZIF-8 composite membrane shows only 56.97% inhibition under light and 9.9% in dark environments, while the pure cellulose acetate membrane exhibits negligible antibacterial activity (<5% inhibition across all conditions).

### 3.7. Practical Application as Food Packaging Film

**Figure 10:**
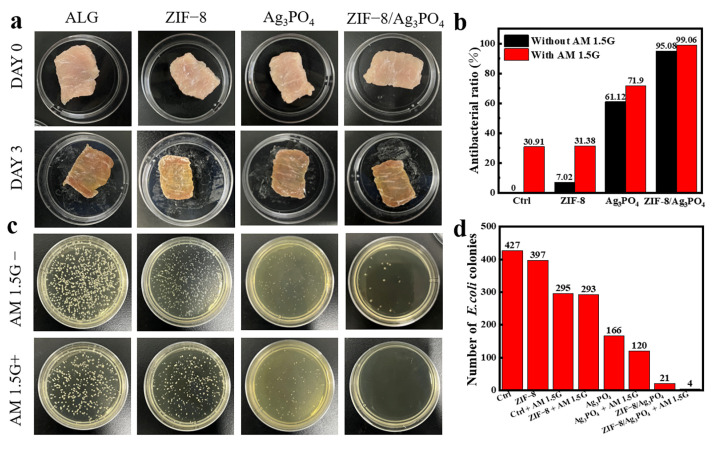
(**a**) Appearance of pork before and after being wrapped with composite membrane materials for three days, (**b**) graph of antibacterial efficiency, (**c**) colonies formed on pork wrapped with composite membrane materials after three days under reaction conditions and simulated sunlight, and (**d**) colony counts.

Fresh pork tenderloin was cut into small pieces and individually wrapped in CAM membranes. After being exposed to room temperature for three days, the meat’s surface color gradually faded, presenting a slightly grayish-brown hue, accompanied by varying degrees of mucus secretion (as depicted in Figure 10a). The colonies present on the pork surface after this period were enumerated using the plate count method. The variation in the sizes and morphologies of the colonies is likely attributed to the diverse bacterial species present on the pork surface in Figure 10c [32]. As illustrated in Figure 10b,d, it is evident that the antibacterial performance under simulated sunlight conditions was superior to that under dark conditions. Specifically, the antibacterial rate of the Ag_3_PO_4_ group reached 71.90%, which can be attributed to the inherent antibacterial properties of Ag^+^.

In contrast, the antibacterial rate of ZIF-8/Ag_3_PO_4_ reached 99.06%, demonstrating superior antibacterial performance compared to single Ag_3_PO_4_ or ZIF-8 materials combined with other substances in the preparation of antibacterial packaging films. In previous research, alginate/κ-carrageenan-based edible films incorporated with clove essential oil exhibited an antibacterial rate of 90.32% [33]. When beef was packaged in polyethylene films coated with chlorine dioxide (1.8%), the total bacterial count on the beef was reduced by approximately 90%; however, this treatment also caused the beef color to change from red to dark green [34]. Comparisons indicate that the composite film also demonstrates good antibacterial performance in practical applications. In addition, we systematically evaluated the production costs of the ZIF-8/Ag_3_PO_4_ composite film based on reagent prices from Macklin Biochemical Co., Ltd. (Shanghai, China) and local suppliers. As detailed in Table S1, the total material cost for fabricating a 15 cm × 25 cm film amounts to CNY 4.145, corresponding to CNY 110.53 per m^2^ (calculated through rigorous unit area normalization).

### 3.8. Mechanism Analysis

The ICP results show that Ag and Zn concentrations in ZIF-8/Ag_3_PO_4_ were 2.675 μg/mL and 1.019 μg/mL, respectively, indicating a molar ratio of Ag_3_PO_4_ to ZIF-8 of 0.53. Additionally, Ag and Zn concentrations in meat packaged with the composite film for three days were 0.615 μg/mL and 0.24 μg/mL, respectively, both within food safety limits.

**Figure 11:**
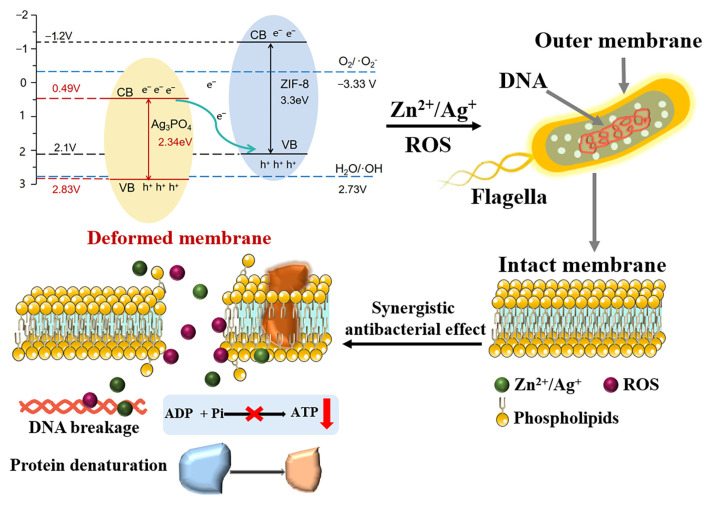
Schematic diagram of the sterilization principle.

Among numerous nano-antibacterial materials, photocatalytic antibacterial materials can utilize the solar spectrum to generate reactive oxygen species for achieving sterilization functions. However, single-phase photocatalytic systems cannot efficiently utilize the solar spectrum due to issues with their bandgap and energy band structure. In this study, the ZIF-8/Ag_3_PO_4_ heterojunction was constructed in Figure 11, which simulates the Z-scheme photosystem in nature and significantly expands the light absorption range, enabling it to make more effective use of both ultraviolet and visible light. Ag_3_PO_4_ has a relatively narrow bandgap of 2.34 eV and can effectively absorb photons in the visible light region to generate electron-hole pairs [35]. Nevertheless, the single Ag_3_PO_4_ system has limited absorption capacity in the UV-vis light region. The introduction of ZIF-8 remarkably expands the light absorption capacity. The Z-scheme heterojunction formed by the two can achieve synergistic absorption under a wide range of solar spectra, thus effectively enhancing the overall photogenerated carrier generation rate of the photocatalytic system and consequently strengthening the overall antibacterial performance of the material under sunlight irradiation.

The Z-scheme photocatalytic architecture critically suppresses carrier recombination, overcoming the inherent limitation of single-phase photocatalysts that suffer from high recombination kinetics, a fundamental barrier to sustained reactive oxygen species (ROS) generation. In the ZIF-8/Ag_3_PO_4_ heterostructure, however, the photogenerated electrons promptly transfer from the conduction band of Ag_3_PO_4_ to the valence band of ZIF-8, while the holes remain in the valence band of Ag_3_PO_4_, accomplishing effective carrier separation. This efficient carrier separation mechanism not only mitigates non-radiative recombination but also prolongs the lifetime of the photogenerated electron-hole pairs, thereby enhancing the generation efficiency of ROS. Consequently, the antibacterial performance of the material is remarkably enhanced. The antibacterial mechanism of the ZIF-8/Ag_3_PO_4_ heterostructure photocatalytic system mainly hinges on the generation of ROS. In the Z-scheme photocatalytic structure, the efficient separation of photogenerated electron-hole pairs facilitates the rapid and stable generation of a series of ROS, such as ·O_2_^−^ and ·OH. These ROS all possess extremely strong oxidative capabilities, enabling them to swiftly damage bacterial cell walls and membranes, leading to cell lysis and enhancing the photocatalytic antibacterial performance [36].

In the ZIF-8/Ag_3_PO_4_ heterostructure photocatalytic system, aside from ROS, the Ag^+^ ions released from Ag_3_PO_4_ also play a significant synergistic role in the antibacterial effect. Ag^+^ ions can bind to proteins and DNA on bacterial cell walls, disrupting bacterial metabolism and reproduction, which consequently leads to cell apoptosis and exhibits broad-spectrum bactericidal activity [37,38,39]. This ionic antibacterial mechanism, in combination with the photocatalytic generation of ROS, constitutes a dual antibacterial mechanism in the ZIF-8/Ag_3_PO_4_ photocatalytic system, endowing the material with excellent bactericidal performance.

## 4. Conclusions

This study developed a novel antibacterial food packaging material by constructing a Z-scheme photocatalytic system based on the heterostructure of ZIF-8 and Ag_3_PO_4_. The system enhances the utilization of the solar spectrum, inhibits electron-hole recombination, and promotes the generation of ·O_2_^−^ and ·OH, thereby achieving synergistic antibacterial performance with Ag^+^. CAM was used as the substrate material, endowing the resulting films with good film-forming properties, biocompatibility, and processability. While maintaining excellent photocatalytic performance, the films also exhibit high permeability, toughness, and mechanical strength. Experimental results demonstrate that the ZIF-8/Ag_3_PO_4_/CAM films achieve a 100% antibacterial rate against *E. coli*, and under simulated sunlight exposure, they achieve an antibacterial rate of 99.06% for meat preservation, showing promising potential for extending food shelf life and reducing food spoilage. Furthermore, the material’s multifunctional design and tunable photocatalytic properties suggest broader applicability in biomedical engineering, precision agriculture, and environmental remediation technologies.

## Data Availability

The original contributions presented in this study are included in the article. Further inquiries can be directed to the corresponding authors.

## Supplementary Materials

The following supporting information can be downloaded at: https://www.mdpi.com/article/10.3390/ma18112544/s1, Figure S1: XPS survey spectra of ZIF-8 and ZIF-8/Ag_3_PO_4_; Figure S2: Elemental distribution analysis of the ZIF-8/Ag_3_PO_4_ heterostructure: (a) EDS composite overlay mapping; (b–e) Individual elemental mappings for (b) Ag, (c) P, (d) O, and (e) Zn; Figure S3: Bacterial live/dead staining images of the ZIF-8 and the Ag_3_PO_4_; Figure S4: (a) *E. coli* bacterial colonies of the powder material under a sample concentration of 19.05 µg/mL; (b) Antibacterial efficiency of the powder material; Figure S5: Composite membranes prepared by dissolving cellulose acetate at mass concentrations of (a) 10% (b) 20% (c) 25% and (d) 30% in acetone; Figure S6: Appearance of CAM membranes for the control group, ZIF-8, Ag_3_PO_4_, and ZIF-8/Ag_3_PO_4_; Table S1: Cost for the synthesis of ZIF-8/Ag_3_PO_4_ composite film.
