# Supplementary material for: Z-Scheme ZIF-8/Ag3PO4 Heterojunction Photocatalyst for High-Performance Antibacterial Food Packaging Films
Source: Materials (Basel). 2025 May 28;18(11):2544. doi: 10.3390/ma18112544 (PMC12155932; doi:10.3390/ma18112544)
Supplement: Supplementary file 1 [file materials-18-02544-s001.zip › materials-3648740-supplementary.pdf]

Supplementary Information

Z-Scheme ZIF-8/Ag_3PO_4 Heterojunction Photocatalyst for High-Performance Antibacterial Food Packaging Films

Qingyang Zhou, Zhuluni Fang, Junyi Wang, Wenbo Zhang, Yihan Liu, Miao Yu, Zhuo Ma, Yunfeng Qiu and Shaoqin Liu

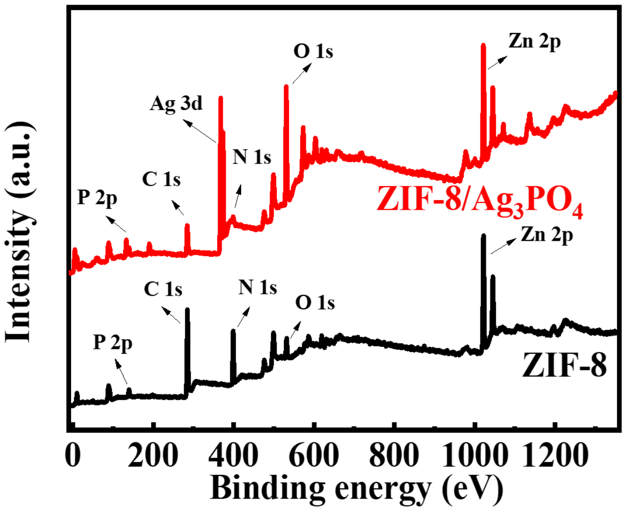

Figure S1. XPS survey spectra of ZIF-8 and ZIF-8/Ag_3PO_4.

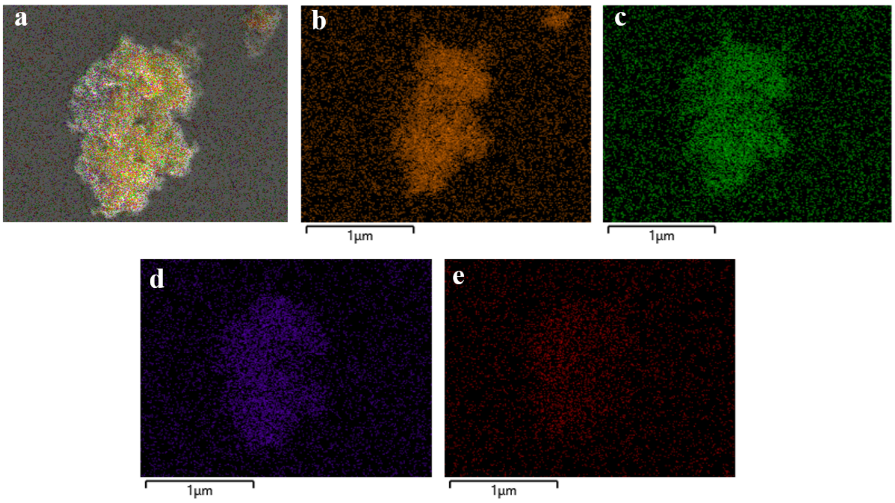

Figure S2. Elemental distribution analysis of the ZIF-8/Ag_3PO_4 heterostructure: (a) EDS composite overlay mapping; (b-e) Individual elemental mappings for (b) Ag, (c) P, (d) O, and (e) Zn.

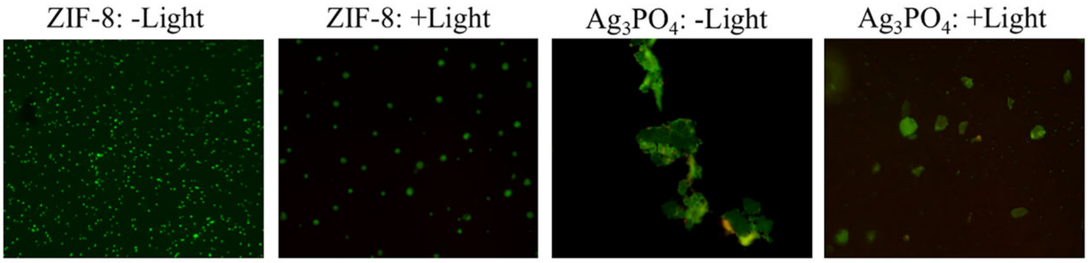

Figure S3. Bacterial live/dead staining images of the ZIF-8 and the Ag_3PO_4.

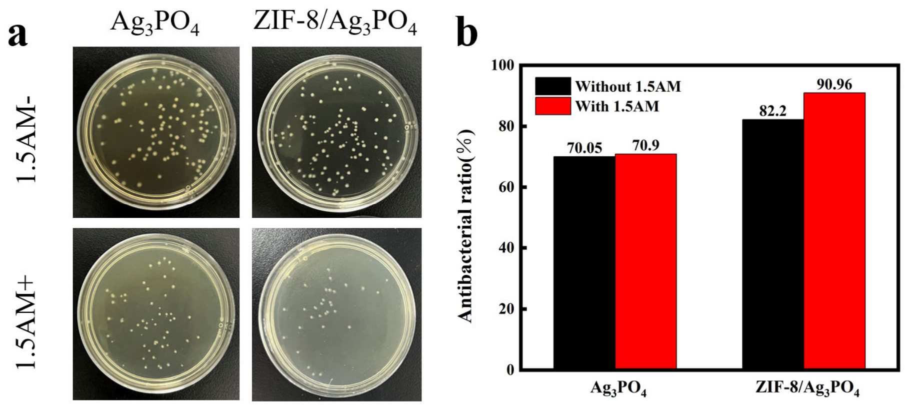

Figure S4. (a) *E. coli* bacterial colonies of the powder material under a sample concentration of 19.05 μg/mL; (b) Antibacterial efficiency of the powder material.

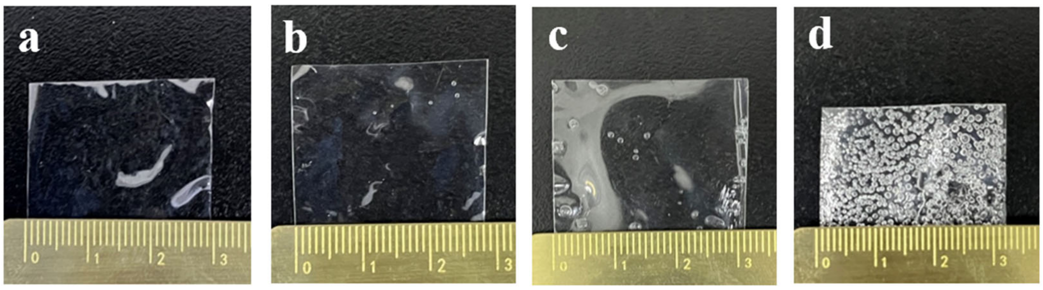

Figure S5. Composite membranes prepared by dissolving cellulose acetate at mass concentrations of (a) 10% (b) 20% (c) 25% and (d) 30% in acetone.

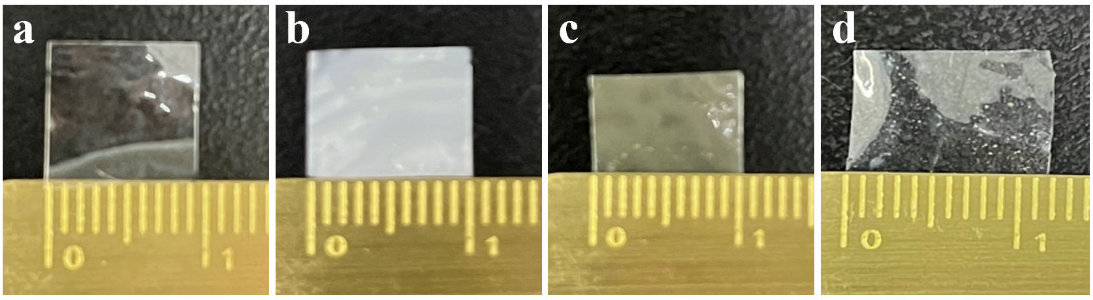

Figure S6. Appearance of CAM membranes for the control group, ZIF-8, Ag_3PO_4, and ZIF-8/Ag_3PO_4.

Table S1. Cost for the synthesis of ZIF-8/Ag_3PO_4 composite film.

Reagent Name	Unit Price (CNY/g or CNY/mL)	Quantity Required per 15 cm × 25 cm Composite Film (g or mL)	Total Cost (CNY)
2-methylimidazole	0.23	0.33 g	0.075
$Zn(NO_3)_2\cdot 6H_2O$	0.26	0.15 g	0.039
PVP	3.20	0.05 g	0.160
$AgNO_3$	5	0.15 g	0.75
NaH_2PO_4	0.14	0.15 g	0.021
CAM	0.30	2.00 g	0.600
CH_3COCH_3	0.25 CNY/mL	10 mL	2.500
15cm × 25cm ZIF-8/Ag_3PO_4 Composite Film	—	—	4.145
1 m^2 ZIF-8/Ag_3PO_4 Composite Film	—	—	110.53
